# The impact of financial incentives on SNAP transactions at mobile produce markets

**DOI:** 10.1186/s12966-021-01093-z

**Published:** 2021-02-08

**Authors:** Pasquale E. Rummo, Reece Lyerly, Jennifer Rose, Yelena Malyuta, Eliza Dexter Cohen, Amy Nunn

**Affiliations:** 1 Department of Population Health, New York University School of Medicine, New York, NY USA; 2 Rhode Island Public Health Institute, Providence, RI USA; 3 Gerald J. and Dorothy R. Friedman School of Nutrition Science and Policy, Tufts University, Boston, MA USA; 4 Wesleyan University, Middletown, CT USA; 5 Center for Health Promotion and Health Equity, School of Public Health, Brown University, Providence, RI USA

**Keywords:** Mobile produce markets, Financial incentives, Fruit and vegetable purchases

## Abstract

**Background:**

Offering financial incentives promotes increases in fruit and vegetable purchases in farmers’ markets and supermarkets. Yet, little is understood about whether food-insecure adults purchase more fruits and vegetables as a result of receiving financial incentives in mobile produce market settings.

**Methods:**

In 2018–2019, Food on the Move provided a 50% discount to customers using Supplemental Nutrition Assistance Program (SNAP) benefits to purchase fruit and vegetables from 16 market locations in Rhode Island (*n* = 412 market occasions). We used mixed multivariable linear regression to estimate the difference in total dollar sales per transaction per month between SNAP transactions and non-SNAP transactions. We also estimated the difference in out-of-pocket dollar sales per transaction per month between SNAP and non-SNAP transactions, less the 50% discount. This reflects the actual amount spent on fresh fruits and vegetables purchased per visit. In both models, we controlled for the number of market sites per month, with fixed effects for quarter and year. We estimated random intercept variance for date of transaction and market site to adjust for clustering.

**Results:**

In 2018–2019, the majority of market transactions (total *n* = 13,165) were SNAP transactions [*n* = 7.988 (63.0%)]. On average, customers spent $17.38 (SD = 16.69) on fruits and vegetables per transaction per month. However, customers using SNAP benefits spent significantly more on FVs per transaction per month [$22.01 (SD = 17.97)] compared to those who did not use SNAP benefits [9.81 (SD = 10.68)] (β = $10.88; 95% CI: 10.18, 11.58). Similarly, out-of-pocket dollar sales per SNAP transaction per month (i.e., less the 50% discount) were significantly higher [$11.42 (SD = 9.44)] relative to non-SNAP transactions [$9.40 (SD = 9.33)] (β = $1.85; 95% CI: 1.44, 2.27).

**Conclusions:**

Financial incentives contributed to higher fruit and vegetable purchases among low-income customers who shop at mobile produce markets by making produce more affordable. Higher spending on fruits and vegetables may promote healthy diet behaviors and reduce chronic disease risk among food-insecure adults.

## Background

Daily fruit and vegetable consumption is essential for maintaining a healthy weight and preventing chronic diseases [1], yet few U.S adults meet daily recommendations, particularly adults of low socioeconomic status [2]. Low-income and minority households are also disproportionately impacted by the burden of food insecurity [3], which is defined as a the economic or social condition of limited or unsafe access to adequate food [4]. These social inequalities may also exacerbate health disparities among non-Hispanic Black and Hispanic households [5]. In the U.S., food insecurity is associated with higher healthcare use and costs and multiple chronic disease risk factors [6–8], as well as limitations in activities of daily living among older adults [9]. The Supplemental Nutrition Assistance Program (SNAP) is the largest federal food assistance program in the U.S., and participants may use their SNAP benefits to purchase any food or beverage, except for alcoholic beverages, pet foods, dietary supplements, hot foods, or non-food items [10]. Participation in SNAP helps alleviate food insecurity, but may not be enough to help low-income adults purchase and consume adequate amounts of fruits and vegetables [11].

Previous studies suggest that cost and access are the main barriers preventing SNAP participants from purchasing and consuming fresh fruits and vegetables [12, 13]. SNAP participants report that consuming healthy foods is cost-prohibitive (relative to the costs of less healthy food items) [12], especially perishable foods such as produce [14]. Qualitative research also suggests that the physical environment influences SNAP participants’ ability to access fresh produce, potentially due to a lack of transportation, time constraints, and few stores selling fruits and vegetables [12]. Mobile produce markets are portable markets designed to mitigate these financial and physical barriers to healthy diet behaviors by selling affordable fruits and vegetables in areas with limited access to fresh produce [15]. A primary goal of mobile produce markets is to support the needs and preferences of underserved communities, including low-income, minority, and older customers. Many mobile produce markets operate out of vans, trucks, or carts, and target multiple community sites in a given day or week. A recent systematic review indicates that mobile produce market use is associated with greater access to and higher consumption of fruits and vegetables, though results are not always consistent [15].

Food on the Move (FOTM) is a mobile produce market based in Providence, RI, and has been operating for over a decade. The FOTM program was informed by the “Live Well, Viva Bien” intervention, which offered fruits and vegetables at a discounted price to residents living in low-income housing sites via bi-weekly produce markets [16]. In spite of the program’s popularity and its effectiveness at markets located in senior housing sites [17], customers still identified cost as a barrier to shopping at the mobile markets. The Rhode Island Public Health Institute (RIPHI) drew upon these findings to create the FOTM program, intentionally targeting senior housing sites for new market locations. In an effort to increase the affordability of produce, FOTM initially offered a matching credit, which allowed SNAP customers to earn $1 on a “Rhody Bucks” gift card for every $1 spent on produce. In response to customer feedback, FOTM transitioned from the “Rhody Bucks” gift card incentive to an automatic 50% discount, thus preserving the match amount while offering a more convenient delivery method.

Financial incentives such as matching credits and discounts may mitigate disparities in diet and obesity risk by making healthy food more affordable [18]. Previous studies have shown that incentive programs at farmers’ markets, such as the Double Up Food Bucks (DUFB) Program, are linked to greater food security and higher fruit and vegetable intake among SNAP participants [19, 20]. Researchers have also tested the impact of financial incentives in supermarket settings, and have found that matching credits and discounts lead to higher spending on fresh fruits and vegetables among eligible customers [21–25]. In a recent study using survey data, more than 75% of SNAP customers at FOTM reported being able to make their benefits last longer by shopping at FOTM [26], potentially due to the incentive.

Mobile produce markets may be an acceptable setting for SNAP incentive programs because they concurrently address physical access barriers to healthy food retailers and incorporate elements of community engagement in their program design. Though mobile produce markets are a promising strategy for mitigating financial barriers to healthy food consumption, few, if any, studies have examined whether SNAP participants purchase more fruit and vegetables as a result of receiving financial incentives in a mobile produce market setting. Similarly, few studies have tested the impact of an immediate 50% discount (versus a matching credit) on fruit and vegetable purchases [22], or analyzed the effects among low-income immigrants living at senior sites.

To address these gaps in the literature, we sought to determine whether an automatic 50% discount on fruits and vegetables was associated with higher spending on fruits and vegetables among those using SNAP benefits at mobile produce markets. We hypothesized that customers using SNAP benefits would spend more on fruits and vegetables per month compared to those not using SNAP benefits in market transactions, as a result of the FOTM program. Based on the success of previous incentive programs in supermarket and farmer’s market settings [19–25], we hypothesized that offering financial incentives would increase fruit and vegetable purchases among customers shopping at mobile produce markets.

## Methods

### Setting and participants

FOTM is an evidence-based mobile produce market operated by RIPHI in Providence, RI. Market staff use a truck and refrigerated trailer to deliver a variety of culturally-appropriate fruits and vegetables to community-based market sites, where market events are held for at least 2 h. FOTM often operates several market events at multiple market sites in 1 day. Self-reported data from our previous work indicates that the majority of FOTM customers are female, Hispanic, over 50 years of age, live alone, earn less than $20,000 a year, currently receive SNAP benefits, and tend to be the primary food shopper for their household [26]. Similar to other mobile produce markets, FOTM is not a full-scale grocer and does not sell non-produce food items.

### Study intervention

FOTM offered an automatic 50% discount incentive to all customers using SNAP benefits to purchase produce at markets, tendered immediately at the point-of-sale. The 50% discount incentive was initially implemented in five market sites in March, 2017, and subsequently all market sites (*n* = 16) in January, 2018 because of its popularity among customers and improved efficiencies with communication and checkout. Between 2018 and 2019, the FOTM program also made two programmatic changes. First, FOTM transitioned all of their sites from monthly to weekly markets, allowing customers to shop more regularly at the markets. This required FOTM to reduce the total number of market sites, selectively keeping the highest performing sites in the transition to monthly markets. Second, FOTM focused on increased outreach and engagement at the remaining market sites, leading to both a larger customer base and more frequent visits. For example, there were an average of 27.9 market events per month at 16 sites in 2018, which shifted to an average of 25.4 market events per month at 8 sites in 2019.

### Data and measures

We used point-of-sale data from January 1, 2018 to May 31, 2019 to examine whether SNAP participants purchased more fruits and vegetables at FOTM mobile produce markets due to an automatic 50% discount. The point-of-sale data includes the date of transaction; total dollar sales; whether a 50% discount was applied; and whether an Electronic Benefit Transaction (EBT) card was used in the transaction. We excluded transactions in which a “Rhody Bucks” gift card was used during the transition to the discount program (*n* = 154), and EBT transactions for which we could not be sure that a 50% discount was applied (*n* = 129).

Our primary outcome was the total dollar sales per transaction per month, inclusive of the value of the 50% discount. This reflects the total amount of fresh fruits and vegetables purchased at FOTM markets per visit in a given month. Our secondary outcome was out-of-pocket dollar sales per transaction, less the 50% discount. This reflects the actual amount spent on fresh fruits and vegetables purchased at FOTM markets per visit in a given month. The independent variable was whether a customer used an EBT card in a transaction (i.e., SNAP benefits). It is possible that some non-SNAP transactions were made by SNAP participants not using their SNAP benefits.

### Statistical analysis

To estimate the difference in total and out-of-pocket dollar sales per transaction between SNAP and non-SNAP transactions, we used mixed multivariable linear regression with a random intercept for date of transaction and market site. In all analyses, we controlled for the number of market sites per month, with fixed effects for quarter and year to account for seasonal and annual effects, respectively. To examine whether programmatic changes (i.e., transition to weekly markets and increased outreach and engagement) contributed to differences in fruit and vegetable purchases in 2019 versus 2018, we also included an interaction term for year and whether an EBT card was used in the transaction. We used a chi-square difference test to assess the significance of the interaction and compare the models with and without the interaction. Statistical significance was defined at the α = 0.05 level. All analyses were performed using R version 4.0.2 (R Core Team, Vienna, Austria, 2019).

## Results

There were 412 markets events at 16 market locations between 2018 and 2019, including 287 market events at 16 market locations in 2018 and 125 market events at 8 market locations during the first half of 2019 (Table 1). Approximately 62% of transactions (*n* = 7988) were made by customers using SNAP benefits across both years, with a small increase in the percentage from 2018 (59.7%) to 2019 (66.2%). The total value of the 50% discount distributed to FOTM customers was $84,606.15 across the study period.

**Table 1:** Total dollar sales^a^ per transaction per month, SNAP and non-SNAP transactions^b^

	All Transactions			SNAP Transactions			Non-SNAP Transactions		
	2018	2019	2018–2019	2018	2019	2018–2019	2018	2019	2018–2019
Market Location (N (%))	16	8	16	16	8	16	16	8	16
Market Event (N (%))	287	125	412	287	125	412	286	124	410
Transactions (N (%))	8266 (64.2)	4616 (35.8)	12,882 (100)	4931 (59.7)	3057 (66.2)	7988 (62.0)	3335 (40.3)	1559 (33.8)	4894 (38.0)
$ Total Sales per Month (mean (SD))	11,006 (3614)	18,355 (3256)	18,653 (10772)	8373 (3046)	15,069 (3492)	14,652 (8649)	2633 (979)	3286 (712)	4002 (2413)
$ Out-of-Pocket Sales per Month (mean (SD))	6924 (2152)	10,822 (1730)	8070 (2699)	4370 (1567)	7756 (1847)	5365 (2252)	2554 (951)	3066 (630)	2705 (883)
$ Sales per Transaction, Total (mean (SD))	15.98 (15.05)	19.88 (19.04)	17.38 (16.69)	20.38 (16.26)	24.65 (20.17)	22.01 (17.97)	9.47 (9.98)	10.54 (12.00)	9.81 (10.68)
$ Sales per Transaction, Out-of-Pocket (mean (SD))	10.05 (8.81)	11.72 (10.42)	10.65 (9.45)	10.63 (8.61)	12.68 (10.53)	11.42 (9.44)	9.19 (9.02)	9.83 (9.94)	9.40 (9.33)
$ 50% Discount per Transaction (mean (SD))	9.74 (8.02)	11.96 (9.95)	10.59 (8.87)	9.74 (8.02)	11.96 (9.95)	10.59 (8.87)	–	–	–

^a^Inclusive of the value of the 50% discount

^b^SNAP transaction defined as a transaction where an EBT card was used

**Fig. 1:**
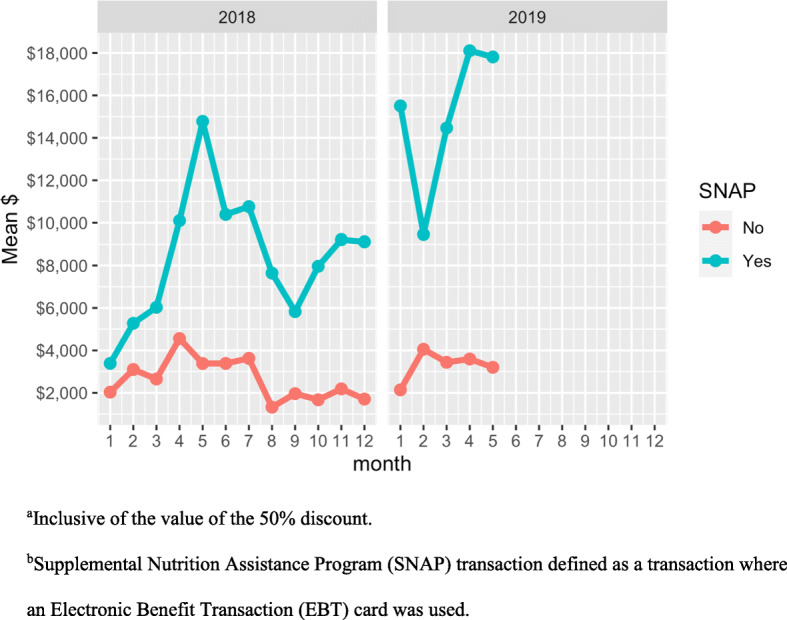
Total dollar sales^a^ per transaction per month, SNAP and non-SNAP transactions^b^

In 2018–2019, total dollar sales per month at all markets was $18,653 (SD = 10,772) (inclusive of the value of the 50% discount). On average, customers spent $17.38 (SD = 16.69) per transaction on fruits and vegetables (Table 1). However, the average total dollar sales per month was higher among those using SNAP benefits [$14,654 (SD = 8649)] than customers not using SNAP benefits [$4002 (SD = 2413)] (Fig. 1). Based on our regression model, spending on fruits and vegetables was significantly higher among SNAP transactions [$22.01 (SD = 17.97)] compared to non-SNAP transactions [$9.81 (SD = 10.68)] (β = $10.88; 95% CI: 10.18, 11.58) (Table 2). Compared to non-SNAP transactions, the average total dollar sales among SNAP transactions was significantly higher in 2019 compared to 2018 (β = $3.09; 95% CI: 1.91, 4.26).

**Table 2:** Mixed multivariable linear regression results, Total dollar sales^a^ and out-of-pocket sales

	Total sales ($)		Out of-pocket sales ($)	
	β	95% CI	β	95% CI
(Intercept)	7.90	4.43, 11.36	9.11	6.69, 11.52
SNAP transaction^b^	10.54	9.85, 11.23	1.86	1.44, 2.27
Number of market sites per month	0.01	−0.10, 0.13	−0.02	−0.10, 0.06
Quarter				
1st	REF	REF	REF	REF
2nd	1.20	−0.24, 2.63	0.40	−0.61, 1.41
3rd	1.42	−0.31, 3.15	0.45	−0.75, 1.66
4th	0.63	−1.21, 2.47	−0.41	−1.70, 0.87
Year				
2018	REF	REF	REF	REF
2019	1.16	−0.45, 2.76	0.38	−0.71, 1.47
SNAP transaction x Year	3.36	2.17, 4.55	1.12	0.41, 1.83

^a^Inclusive of the value of the 50% discount

^b^Supplemental Nutrition Assistance Program (SNAP) transaction defined as a transaction where an Electronic Benefit Transaction (EBT) card was used

**Fig. 2:**
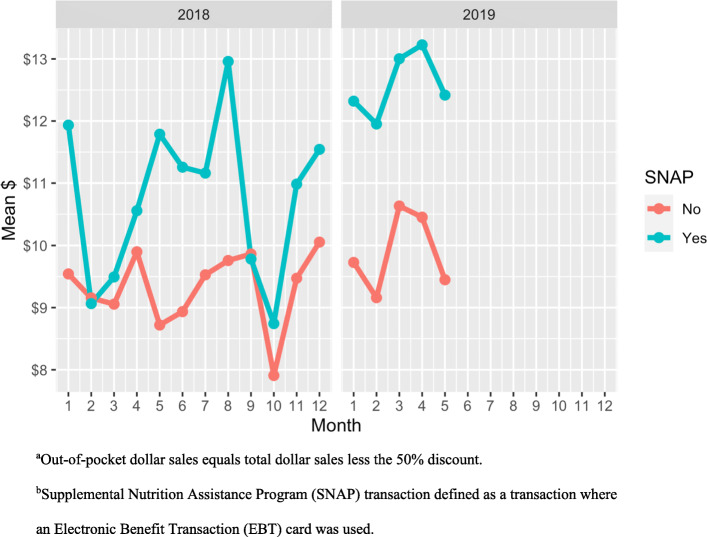
Out of-pocket dollar sales^a^ per transaction per month, SNAP and non-SNAP transactions^b^

**Fig. 3:**
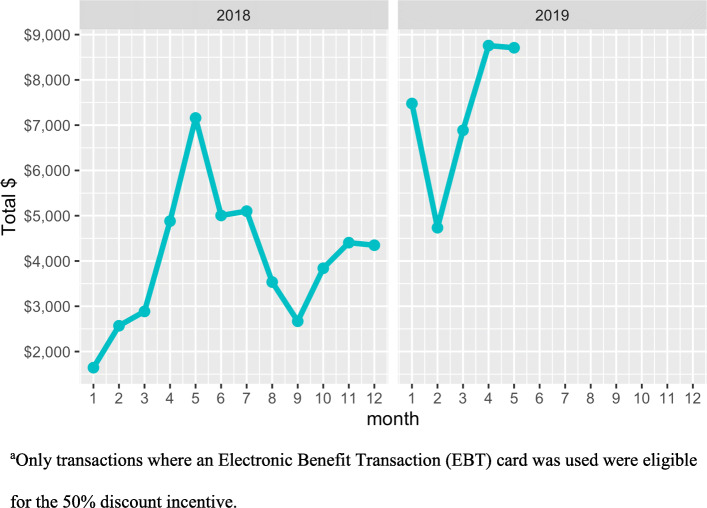
Total 50% discount incentives distributed^a^ per transaction per month

The average out-of-pocket dollar sales per SNAP transaction per month [$5365 (SD = 2252)] was higher than non-SNAP transactions [$2705 (SD = 883)] (Table 1). Similarly, out-of-pocket dollar sales per transaction was significantly higher among SNAP transactions [$11.42 (SD = 9.44)] relative to non-SNAP transactions [$9.40 (SD = 9.33)] (β = $1.85; 95% CI: 1.44, 2.27) (Fig. 2). The out-of-pocket dollar sales per transaction was also significantly higher in 2019 compared to 2018 (β = $1.46; 95% CI: 0.62, 2.29) (Table 2), which was paralleled by the increase in total incentives distributed per month in 2019 (Fig. 3).

## Discussion

In 2018–2019, the FOTM program offered a 50% discount on fresh produce to customers using SNAP benefits to shop at their mobile produce markets. The incentive was associated with significantly higher spending on fruits and vegetables in SNAP (versus non-SNAP) transactions, with greater effects in 2019. Indeed, total dollar sales and out-of-pocket dollar sales were both higher in SNAP transactions; this suggests customers using SNAP benefits purchased a higher quantity of fruits and vegetables and spent more fruits and vegetables at FOTM markets than customers not using SNAP benefits. These results suggest that the FOTM program is effective in promoting fruit and vegetables purchases among low-income consumers, most of whom are low-income Hispanic/Latino populations and older adults.

Fruit and vegetable incentives address cost barriers by increasing a customer’s financial resources for purchasing healthy food items [27]. For example, we recently analyzed responses from a survey of 314 customers shopping at FOTM markets in 2018, and found that that the 50% discount was associated with higher self-reported consumption of fruits and vegetables among SNAP (versus non-SNAP) customers [26]. The survey also showed that 75.6% of SNAP customers were able to make their benefits last longer by shopping at FOTM, which may explain why SNAP customers spent more out-of-pocket than non-SNAP customers. For these reasons, we posit that higher spending on fruits and vegetables observed in the current study may correspond with higher consumption of fruits and vegetables by increasing the affordability of fresh produce for customers receiving food assistance. Given the relationship between fruit and vegetable consumption and chronic disease prevention [1], it is also possible that financial incentives for produce may be an effective tool in mitigating economic disparities in poor health outcomes.

Our findings are consistent with previous assessments of similar programs. Prior research has shown that offering subsidies and discounts for produce increases fruit and vegetable purchases among SNAP participants shopping in supermarkets and farmer markets [19–25]. The most prominent example is the Healthy Incentives Pilot (HIP), which offered SNAP participants an incentive of 30 cents for every dollar of SNAP benefits that they spent on eligible fruits and vegetables in participating supermarkets [21]. Spending on eligible fruit and vegetables increased by 11% and consumption of fruit and vegetables increased by 26% in HIP households compared to non-HIP households. Our findings demonstrate that financial incentives are also effective in promoting fruit and vegetable purchases in mobile produce markets, which have the added benefit of improving access to healthy food in food deserts and among vulnerable populations [15]. In addition to main effects, we also observed that the difference in dollar sales was relatively higher in 2019 than 2018, suggesting that the impact of the incentive became more pronounced over time, potentially due to the transition to weekly markets and increased outreach and engagement. This evidence supports the expansion of the FOTM program, including the development of similar programs in other locations.

Programmatically, we found that the 50% discount model was no more challenging to implement than the matching gift card model, and it was more popular among FOTM customers. Thus, transitioning to a discount model is feasible for programs responsible for distributing incentives independently (i.e., no additional EBT technology). However, one of the challenges with the success of the 50% discount model is that FOTM’s distribution of SNAP incentives greatly outpaced the funding capacity of RIPHI, threatening the long-term sustainability of the program. In 2019, FOTM distributed its entire annual Food Insecurity Nutrition Incentive (FINI) budget for SNAP incentives in less than 5 months, necessitating additional scaling back of the program. Other similar programs, such as Massachusetts HIP, have had similar challenges related to demand and greatly exceeding funding capacity. While this growth is successful from a public health perspective, it puts an undue burden on non-profit organizations that depend on grant funding to sustain their incentive programs. One strategy to support these efforts is to eliminate the non-federal match requirement and increase the funding allotment for the Gus Schumacher Nutrition Incentive Program (formerly FINI).

This study has several limitations, including a lack of baseline data and food intake data. The lack of baseline data prevented us from characterizing purchases in the pre-intervention period, and thus we cannot know whether trends in produce purchases between SNAP and non-SNAP transactions were constant over time. This undermines the internal validity of our analysis and our ability to establish a causal relationship between the 50% discount and fruit and vegetable purchases. The lack of food intake data does not allow us to explicitly link individual purchases with individual consumption, though our previous work shows that self-reported consumption of fruits and vegetables was also higher in our target population [26]. Another limitation was a lack of contextual information about non-SNAP transactions, which prevented us from comparing purchases between SNAP and non-SNAP customers (vs. transactions). This analysis would have allowed us to capture changes in fruit and vegetable purchases due to both the incentive and other factors that might encourage SNAP customers to purchase more fruits and vegetables when not using an EBT card (e.g., changes in food preferences). Though mobile produce markets and incentives may be feasible and effective solutions to food insecurity in many localities, our focus on a specific state and minority and older adults also reduces the generalizability of our findings. To our knowledge, however, our study is the first assessment of the impact of financial incentives on fruit and vegetable purchases in a mobile produce market setting (vs. farmers’ markets and supermarkets). In addition to innovation, our key strengths include using point-of-sale data, a focus on both total and out-of-pocket spending, and a nuanced approach to assessing temporal differences in program effectiveness.

Mobile produce markets may improve food access among low-income adults living in senior sites and minority communities [15]. Our study shows that offering financial incentives may also contribute to higher fruit and vegetable purchases among food-insecure adults who shop at mobile produce markets by making produce more affordable. Higher spending on fruits and vegetables may mitigate economic disparities in food insecurity, and thus chronic disease risk among minority and older adults receiving SNAP benefits. In the future, researchers should explore whether the type of financial incentive matters (e.g., matching credit, 50% discount) and other ways to optimize the mobile produce markets.

## Data Availability

The datasets used and/or analyzed during the current study are available from the corresponding author on reasonable request.

## Abbreviations

SNAP: Supplemental Nutrition Assistance Program
FOTM: Food on the Move
RIPHI: Rhode Island Public Health Institute
FINI: Food Insecurity Nutrition Incentive
